# Research advances in regulation and genetic engineering of floral scents

**DOI:** 10.5511/plantbiotechnology.24.0312a

**Published:** 2024-06-25

**Authors:** Takao Koeduka

**Affiliations:** 1 Graduate School of Sciences and Technology for Innovation, Yamaguchi University

**Keywords:** floral scents, metabolic engineering, petunia, rhythmic emission, volatile benzenoids/phenylpropanoids

## Abstract

Floral scents play important ecological roles because they attract pollinators and seed-dispersers. Historically, humans have used plant volatiles, including floral scents, as food additives, cosmetic products, and medicines. Floral scent formation and emissions are sometimes considerably affected by environmental and climatic conditions. Both enzymes and genes involved in floral scent biosynthesis have been consistently identified, and have provided insights into the potential of metabolic engineering of floral scents. This review summarizes recent studies on various aspects of floral scent biosynthesis and emission, including biosynthetic enzymes and genetic engineering. The findings ultimately show that the metabolic pathways of floral volatiles may be regulated by a more complex system than previously thought.

## Introduction

**Figure 1:**
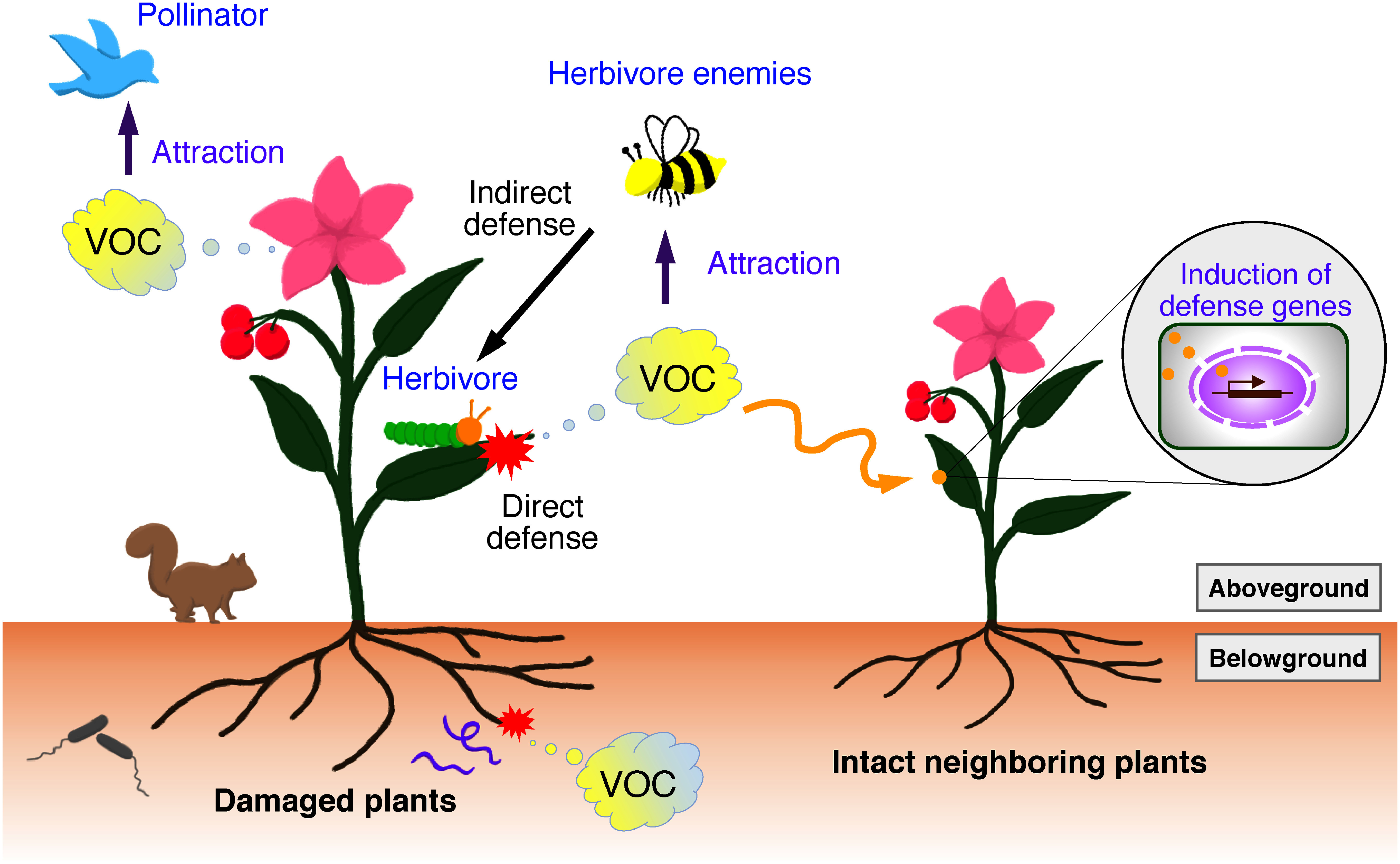
Figure 1. Functions of plant volatiles. Plants produce volatile organic compounds (VOCs), including terpenoids, fatty acid derivatives, and benzenoids/phenylpropanoids, for chemical defense against herbivore attacks both above and below the ground. Volatile compounds produced by floral tissues or through feeding damage also function as signaling molecules to attract pollinators and herbivore enemies, predators, and parasitoids. Neighboring intact plants perceive the volatiles released from damaged plants as warning signals that trigger the induction of defense genes against upcoming stresses.

Plants have evolved the ability to produce a diverse array of specialized metabolites to adapt to their surroundings. In particular, plants produce floral volatiles, which are lipophilic molecules with low boiling points and high vapor pressures at ambient temperatures. Unlike non-volatile metabolites, plant volatiles, including floral scents, are considered as airborne signals for attracting pollinators, seed dispersers, and other beneficial animals and microorganisms, and serve as communication molecules in plant-plant interactions (Figure 1). Volatile compounds emitted from flowers are important features of floral scents and vary widely among flowering plants. In addition to their ecological significance, the differences and abundances of floral scents affect the agronomic and commercial value of horticultural plants. Based on their origin and biosynthesis, floral volatiles can be classified into terpenoids, fatty acid derivatives, and benzenoids/phenylpropanoids.

Over the last several decades, numerous studies have expanded our understanding of the function, composition, biosynthesis, and regulation of floral scents. The discovery of biosynthetic genes has allowed the modification of volatile compounds via genetic engineering to improve the aroma quality of flowers. Although metabolic engineering has been mostly successful for the desired modification of floral scents, several experiments have resulted in unpredictable metabolic consequences owing to further endogenous metabolic modification of the original end products or metabolic feedback inhibition. This review highlights the latest findings in floral scent research and discusses recent efforts to modify floral scent profiles using genetic engineering.

## Floral volatiles and their biosynthetic pathways

### Biosynthesis of volatile terpenoids

**Figure 2:**
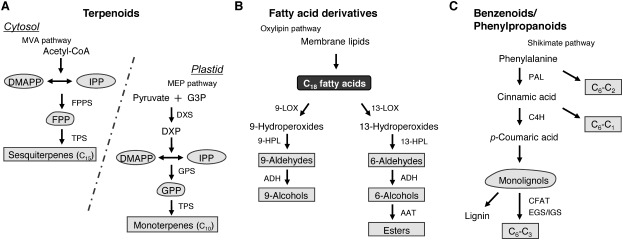
Figure 2. Biosynthetic pathways of the major volatile compound classes in plants. (A) Synthesis of volatile terpenoids. Terpenoid precursors, acetyl-CoA and pyruvate, enter the cytosolic mevalonate (MVA) pathway to produce sesquiterpenes or the plastidial methylerythritol phosphate (MEP) pathway to produce monoterpenes. The enzymes and intermediates of both pathways are shown: DMAPP, dimethylallyl diphosphate; IPP, isopentenyl diphosphate; FPP, farnesyl diphosphate; GPP, geranyl diphosphate; FPPS, farnesyl diphosphate synthase; TPS, terpene synthase; DXP, 1-deoxy-D-xylulose-5-phosphate; DXS, DXP synthase; GPS, geranyl diphosphate synthase. (B) Synthesis of fatty acid-derived volatiles. Fatty acid precursors, such as linoleic and linolenic acid, enter the oxylipin pathway, after which lipoxygenase (LOX) oxidizes them to 9- and 13-hydroperoxides, which are further converted to short-chain volatiles by hydroperoxide lyase (HPL), alcohol dehydrogenase (ADH), and alcohol acyltransferase (AAT). (C) Synthesis of volatile benzenoids/phenylpropanoids. Phenylalanine is produced in the plastids through the shikimate pathway. All volatile benzenoids/phenylpropanoids are produced in the cytosol and peroxisomes. PAL, phenylalanine ammonia lyase; C4H, cinnamate 4-hydroxylase; CFAT, coniferyl alcohol acyltransferase; EGS, eugenol synthase; IGS, isoeugenol synthase; C_6_-C_1_, benzenoids; C_6_-C_2_, phenylpropanoid-related compounds; C_6_-C_3_, phenylpropenes.

Volatile terpenoids, primarily monoterpenes (C_10_) and sesquiterpenes (C_15_), constitute the largest class of floral scents. All volatile terpenoids are produced from the five-carbon units, isopentenyl diphosphate (IPP; C_5_) and dimethylallyl diphosphate (DMAPP; C_5_), which are derived from two different biosynthetic pathways, the cytosolic mevalonate (MVA) pathway and plastidial methylerythritol phosphate (MEP) pathway (Figure 2A). In the cytosol, the MVA pathway begins with the condensation of acetyl-CoA to form MVA, followed by the synthesis of IPP and DMAPP. Subsequently, condensation of DMAPP with two IPP molecules by prenyltransferase forms farnesyl diphosphate (FPP; C_15_). In contrast, the MEP pathway, localized in the plastids, starts with the condensations of pyruvate and glyceraldehyde 3-phosphate to form 1-deoxy-D-xylulose-5-phosphate (DXP), which is catalyzed by DXP synthase. These reactions synthesize IPP and DMAPP, which are then used by prenyltransferases in condensation reactions to produce geranyl diphosphate (GPP; C_10_). After the formation of prenyl diphosphates GPP and FPP, various monoterpenes and sesquiterpenes are biosynthesized by terpene synthases.

### Biosynthesis of volatile fatty acid derivatives

Volatile fatty acid derivatives, including short-chain alcohols and aldehydes, are a primary class of floral volatiles, which are produced from membrane lipids via the oxylipin pathway through an oxygenation reaction by lipoxygenase (Figure 2B). Lipoxygenase is the first enzyme responsible for the formation of volatile fatty acid derivatives. Lipoxygenase recognizes the *1Z*,*4Z*-pentadiene moiety of C_18_ fatty acids (linoleic acid and linolenic acid) and adds molecular oxygen at the C-9 or C-13 position to generate 9- or 13-hydroperoxide of the fatty acids, respectively. Subsequently, the fatty acid hydroperoxides are cleaved by hydroperoxide lyase to form C9 short-chain aldehydes, such as (*Z*,*Z*)-3,6-nonadienal or (*Z*)-3-nonenal, or C6 short-chain aldehydes, such as (*Z*)-3-hexenal or hexanal, depending on the C18 substrate. These short-chain aldehydes are common constituents of floral and green-leaf volatiles and can be converted to their corresponding alcohols or acetyl esters by alcohol dehydrogenase or acyltransferase, respectively.

### Biosynthesis of volatile benzenoids/phenylpropanoids

Phenylalanine (Phe)-derived compounds, designated as benzenoids and phenylpropanoids, are a primary groups of volatile organic compounds in plants and are biosynthesized via several branched pathways (Figure 2C). All these compounds maintain a six-carbon aromatic ring derived from Phe and can be generally classified into three classes (C_6_-C_1_, C_6_-C_2_, and C_6_-C_3_ compounds) based on the length of their side chains.

To form volatile benzenoids and phenylpropanoids, the first step is catalyzed by deamination with phenylalanine ammonia-lyase (PAL), which produces cinnamic acid from Phe. The formation of volatile benzenoids (C_6_-C_1_) from cinnamic acid requires two-carbon shortening of the propyl side chain via two alternative pathways, the β-oxidative and non-β-oxidative pathways (Widhalm and Dudareva 2015).

Of the phenylpropanoids with a C_6_-C_3_ structure as the basic skeleton, only those that are reduced at the C9-position are volatiles, and are generally called phenylpropenes. The biosynthetic pathway of volatile phenylpropenes, such as eugenol and isoeugenol, has been elucidated in *Clarkia breweri* and petunia flowers (Koeduka et al. 2008). Moreover, the initial biosynthetic steps up to the production of monolignols, such as *p*-coumaryl alcohol and coniferyl alcohol, which are produced from cinnamic acid, are shared with lignin biosynthesis. To eliminate oxygen at the C9 position, monolignols are converted to their acetyl esters by coniferyl alcohol acetyltransferase (Dexter et al. 2007) and then reduced to eugenol or isoeugenol by eugenol or isoeugenol synthase, respectively (Koeduka et al. 2006).

Compared to C_6_-C_1_ and C_6_-C_3_ compounds, phenylpropanoid-related C_6_-C_2_ compounds, such as phenylethyl alcohol and phenylacetaldehyde, are biosynthesized directly from Phe via a PAL-independent route. In petunia, rose, and loquat flowers, the bifunctional enzyme phenylacetaldehyde synthase, which catalyzes the decarboxylation amine oxidation reaction, produces phenylacetaldehyde from Phe (Kaminaga et al. 2006; Koeduka et al. 2017). However, an alternative route has also been reported for rose flowers (Hirata et al. 2016). Phe is first converted to phenylpyruvate by an aromatic amino acid aminotransferase, followed by the formation of phenylacetaldehyde by phenylpyruvate decarboxylase.

## Regulation of floral scent production

### Temporal regulation

In general, during the flower lifespan, the production and emission of floral scents are regulated during developmental stages. In the flowers of most scent-emitting plants, volatile emission increases depending on the flowering stage, reaches its peak when the flowers are opened, and then gradually decreases or stops when pollination is completed. For example, the flowers of loquats belonging to the Rosaceae family increase the production of volatile benzenoids, including *p*-anisaldehyde, *p*-anisalcohol, and methyl *p*-methoxybenzoate, as the flowers open (Koeduka et al. 2016). Tuberose flowers (*Agave amica*) exhibit similar behavior; the emission of terpenoid and benzenoid volatiles begins as soon as the flowers open and reaches a peak after flowering (Kutty et al. 2021). The emission patterns of floral scents correlated considerably with the expression patterns of their biosynthetic genes, loquat MBMT and tuberose BEBT. In petunia flowers, gibberellins negatively regulate the production of volatile benzenoids/phenylpropanoids in the early developmental stages, and the volatile levels increase depending on floral development with a reduction in gibberellin levels (Ravid et al. 2017). During senescence or when flowers are pollinated, ethylene increases in floral organs, which reduces floral volatile production through suppression of the biosynthetic gene expression; this is performed by ethylene response factor 6, which interacts with transcription factors regulating biosynthetic genes of volatile compounds (Liu et al. 2017; Underwood et al. 2005; Wang et al. 2013).

To successfully attract pollinators, some plant species emit floral scents with a daily light/dark cycle as well as their characteristic floral scent composition. The rhythmic emission of floral scents is thought to have evolved adaptively such that plants produce floral volatiles when their primary pollinators are active and controlled by a circadian clock or photoperiod. For example, petunia and wild tobacco (*Nicotiana sylvestris*) flowers, which attract nocturnal moths as pollinators, emit a specific set of volatile compounds at night (Fenske and Imaizumi 2016; Loughrin et al. 1990). In contrast, snapdragons, which are pollinated by diurnal bumblebees, exhibit diurnal rhythmicity in scent emissions (Dudareva et al. 2000). Rhythmic volatile emission patterns are regulated at the transcriptional level by genes responsible for volatile compound production. Recently, isolation of the petunia clock transcription factor PhLHY was reported (Fenske et al. 2015). PhLHY regulates the timing of volatile emissions by controlling the expression profiles of volatile benzenoid/phenylpropanoid pathway genes.

Although rhythmic emission allows plants to save carbon resources during times when their pollinators are inactive, some plants emit a constant level of floral scent without distinction between day and night. Investigations of *Rosa hybrida* cv. Piaget revealed that the total amount of volatiles emitted did not change over a day/night cycle throughout the year. However, a regulatory mechanism was found in which multiple biosynthetic pathways producing a single scent compound, phenylethyl alcohol, are activated in response to seasonal temperature changes to maintain constant volatile emission (Hirata et al. 2016).

### Spatial regulation

Among the plant organs, flowers produce the most diverse and abundant volatile compounds. Within flowers, the petals are the primary site of production and emission of floral scent, which often depends on the specific expression of the biosynthetic genes of volatile compounds, although other parts, such as the pistil and anthers, also contribute to volatile production. In *Clarkia breweri* flowers, the highest activity levels of the enzymes BEAT and SAMT, which are responsible for the formation of benzyl acetate and methyl salicylate, respectively, were found in the petals (Dudareva et al. 1998).

In petunia flowers, the adaxial epidermis in the corolla limb, particularly the petal base, emits significantly higher levels of volatiles than the abaxial epidermis (Skaliter et al. 2021). Similar spatial emission patterns were observed in carnation (*Daianthus caryophyllus*) and marguerite daisy (*Argyranthumum frutescens*). Tissue- and organ-specific scent emissions are regulated by the ABC transporter in petunia flowers. The expression levels of the plasma-membrane transporter PhABCG1 in adaxial petals were higher than those on the abaxial side, establishing the spatial emission patterns on the epidermal sides (Adebesin et al. 2017; Skaliter et al. 2021).

Floral volatiles are emitted from highly specific tissues and cells in scent-emitting flowers. For example, in snapdragon flowers, the floral volatile methylbenzoate is mostly produced and emitted from the upper and lower petal lobes (Kolosova et al. 2001). Immunofluorescence analysis showed that the enzyme BAMT, which is responsible for methyl benzoate formation, was predominantly localized in the conical cells of the inner epidermal cells, and to a much lesser extent in the outer epidermal cells.

### Transcriptional factors

Transcription factors play important roles in regulating the expression of biosynthetic genes controlling the production of plant-specialized metabolites, including floral scent. Among the transcription factors, MYB is the most well-known in plants. To date, several MYB transcription factors have been reported to regulate floral scent production and emission. The first transcription factors to be characterized was ODORANT1 (ODO1), which is found in *Petunia hybrida* (Verdonk et al. 2005). ODO1 is highly expressed in the petals during early development of petunia flowers and activates the shikimate and phenylpropanoid pathway genes, thus regulating the substrate supply for volatile benzenoid/phenylpropanoid compounds (Boersma et al. 2022; Verdonk et al. 2005). In addition to biosynthetic genes, ODO1 controls floral scent emission by activating the PhABCG1 transporter, which is localized in the plasma membrane and transports volatile benzenoid/phenylpropanoid compounds (Adebesin et al. 2017).

Furthermore, EOBI and EOBII are MYB transcription factors that are also involved in floral scent production in petunia. EOBII directly activates both ODO1 and EOBI transcripts and positively regulates the expression of key genes involved in volatile benzenoid/phenylpropanoid biosynthesis, such as PAL, CFAT, and IGS (Spitzer-Rimon et al. 2010). In contrast, EOBI was found to be downstream of EOBII and upregulated ODO1 and volatile benzenoid/phenylpropanoid biosynthetic genes, such as IGS and BSMT (Spitzer-Rimon et al. 2012). Although the aforementioned transcription factors regulate shikimate pathway genes or volatile benzenoid/phenylpropanoid biosynthetic genes, only one transcription factor, PhMYB4, was found to repress PhC4H transcription and had no direct effect on the expression of the biosynthetic pathway genes and other transcription factors. PhC4H is involved in *p*-coumaric acid production in petunia and induces a flux of phenylpropanoid volatiles, thereby indirectly controlling the balance of floral scent production between the compounds derived from cinnamic acid and *p*-coumaric acid (Colquhoun et al. 2011).

## Metabolic engineering of floral scent

Researchers have attempted to modify floral scent through metabolic engineering, mainly using petunia and tobacco, which are model plants for studying floral volatiles (Table 1). One possible strategy is to modify the existing pathway or introduce a new branch into the pathway by redirecting metabolic fluxes, either by the overexpression of existing enzymes or heterologous expression of enzymes directly involved in volatile biosynthesis. For instance, constitutive overexpression of three lemon monoterpene synthases in tobacco flowers led to the emission of β-pinene, limonene, and γ-terpinene, which were not detected in the wild-type control (Lücker et al. 2004). In transgenic petunias and carnations overexpressing linalool synthase from *Clarkia breweri*, linalool or its derivatives, linalool oxide and glycoside, were observed (Lavy et al. 2002; Lücker et al. 2001). Overexpression of alcohol acetyltransferase in *Rosa hybrida* (Guterman et al. 2006) and phenylacetaldehyde reductase in tomatoes (Tieman et al. 2007) also led to substantially enhanced production of benzyl- and phenylethyl-acetate, and phenylethyl alcohol, respectively.

**Table 1:** Table 1. Genes used in the metabolic engineering of floral volatiles.

Gene	Origin	Engineered species	Changes in volatile profiles	References
Overexpression				
γ-Terpinene synthase, Limonene synthase, β-Pinene synthase	*Citrus limon*	Tobacco	γ-Terpinene ↑, Limonene ↑, β-Pinene ↑	Lücker et al. (2004)
LIS	*Clarkia breweri*	*Petunia*	Linalool oxides ↑	Lücker et al. (2001)
LIS	*Clarkia breweri*	Carnation	(*S*)-Linalool ↑, Linalool glycosides ↑	Lavy et al. (2002)
AAT	Rose	*Petunia*	Linalool oxides ↑	Guterman et al. (2006)
PAR	Tomato	*Petunia*	Phenylethyl alcohol ↑	Tieman et al. (2007)
PAP1	*Arabidopsis thaliana*	Rose	Eugenol ↑, Germacrene D ↑, β-ionone ↑	Zvi et al. (2012)
PAP1	*Arabidopsis thaliana*	*Petunia*	Benzaldehyde ↑, Vanillin ↑, Eugenol ↑	Zvi et al. (2008), Cna’ani et al. (2015)
AroG	*Escherichia coli*	*Petunia*	Eugenol ↑, Benzaldehyde ↑, Phenylacetaldehyde ↑	Oliva et al. (2015)
PheA	*Escherichia coli*	*Petunia*	Phe-derived volatiles ↑	Oliva et al. (2017)
pCAT	*Petunia*	*Petunia*	Phe-derived volatiles ↑	Widhalm et al. (2015)
BAS, RZS1	Rhubarb, Raspberry	Tobacco	Raspberry ketone ↑, Raspberry ketone glycoside ↑, Rhododenol ↑, Rhododenol glycoside↑	Koeduka et al. (2021)
RNAi suppression				
C3H	*Petunia*	*Petunia*	Phe-derived volatiles ↓	Kim et al. (2019)
TE	*Petunia*	*Petunia*	Benzylbenzoate ↑, Phenylethylbenzoate ↑	Adebesin et al. (2018)
CNL	*Petunia*	*Petunia*	Benzylbenzoate ↓, Phenylethylbenzoate ↓, Methylbenzoate ↓	Klempien et al. (2012)
CSE	*Petunia*	*Petunia*	Phe-derived volatiles ↓	Kim et al. (2023)
CHD	*Petunia*	*Petunia*	Benzylbenzoate ↓, Benzaldehyde ↓, Methylbenzoate ↓	Qualley et al. (2012)
KAT	*Petunia*	*Petunia*	Phe-derived volatiles ↓	Moerkercke et al. (2009)
BSMT	*Petunia*	*Petunia*	Methylbenzoate ↓	Underwood et al. (2005)
PAAS	*Petunia*	*Petunia*	Phenylacetaldehyde ↓	Kaminaga et al. (2006)
BPBT	*Petunia*	*Petunia*	Benzylbenzoate ↓	Orlova et al. (2006)
CFAT	*Petunia*	*Petunia*	Isoeugenol ↓	Dexter et al. (2007)

Gene abbreviations: LIS, linalool synthase; AAT, alcohol acetyl transferase; PAR, phenylacetaldehyde reductase; PAP1, production of anthocyanin pigment 1; AroG, 3-deoxy-D-arabino-heptulosonate 7-phosphate (DAHP) synthase; PheA, chorismate mutase *p*-prephenate dehydratase; pCAT, plastidial cationic amino-acid transporter; BAS, benzalacetone synthase; RZS1, raspberry ketone/zingerone synthase 1; C3H, *p*-coumarate 3-hydroxylase; TE, thioesterase; CNL, cinnamate-CoA ligase; CSE, caffeoyl shikimate esterase; CHD, cinnamoyl-CoA hydratase/dehydrogenase; KAT, 3-ketoacyl thiolase; BSMT, benzoic acid/salicylic acid carboxyl methyltransferase; PAAS, phenylacetaldehyde synthase; BPBT, benzyl alcohol/phenylethanol benzoyl transferase; CFAT, coniferyl alcohol acyltransferase.

The outcomes of these modifications are also observed upon improving the availability of the required substrates. Another strategy for changing floral scent is the incorporation of a chimeric gene encoding bacterial feedback-insensitive *DAHPS* and bi-functional chorismate mutase (CM)/prephenate dehydratase (termed *AroG* and *PheA*, respectively) into transgenic petunia, thereby stimulating the synthesis of substrate precursors, subsequently allowing additional production of various Phe-derived volatile benzenoids/phenylpropanoids (Oliva et al. 2015, 2017). In addition, floral scent modification has been accomplished by changing endogenous substrate levels using transporters. Overexpression of a plastidial cationic amino acid transporter PhpCAT, which exports plastidial phenylalanine, increased the production of Phe-derived volatile benzenoids/phenylpropanoids (Widhalm et al. 2015).

Another approach towards floral scent modification involves the introduction of transcription factors, which regulate scent-producing enzymes, in host plants. Rose flowers introduced with *Arabidopsis* PRODUCTION OF ANTHOCYANIN PIGMENT1 (PAP1), a transcriptional activator of the phenylpropanoid pathway, exhibited elevated production of phenylpropanoid-derived color and scent compounds without undesirable growth inhibition (Zvi et al. 2012). Petunia flowers overexpressing PAP1 also displayed increased floral scent levels, such as benzaldehyde and vanillin, with anthocyanin accumulation (Cna’ani et al. 2015; Zvi et al. 2008). Thus, the transcription factors that regulate volatile biosynthesis are a suitable target for the metabolic engineering of floral scents.

**Figure 3:**
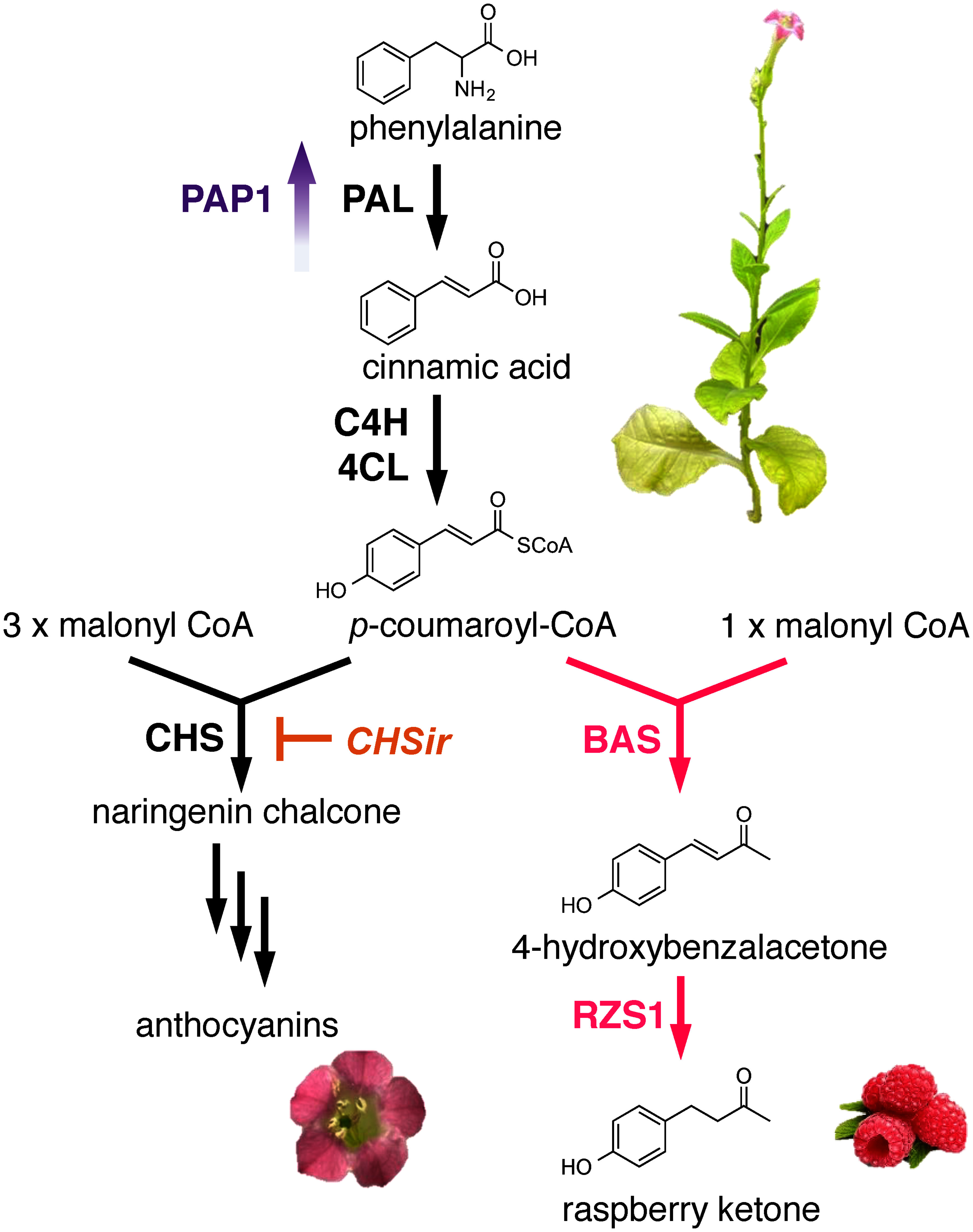
Figure 3. Production of raspberry ketone via the metabolic engineering of phenylpropanoid pathway in *Nicotiana tabacum*. Overexpression of *BAS* and *RZS1* generate raspberry ketone (pink) in transgenic tobacco. Co-expression of the transcription factor *PAP1* (purple) and RNAi-mediated downregulation of *chalcone synthase* (CHSir; orange) at key branchpoints to ensure carbon flow directionality enhanced the levels of raspberry ketone.

An alternative approach for the modification of floral scents is the reduction or elimination of volatile compounds. Transgenic petunias with reduced levels of methylbenzoate (Underwood et al. 2005), phenylacetaldehyde (Kaminaga et al. 2006), benzylbenzoate (Orlova et al. 2006), and isoeugenol (Dexter et al. 2007) were obtained via RNAi downregulation of a single gene, BSMT, PAAS, BPBT, or CFAT, respectively (Table 1). Recently, the transcript levels of multiple genes have been altered through genetic modification to optimize metabolic fluxes of floral volatiles. In addition to benzalacetone synthase and raspberry ketone/zingerone synthase 1, flavonoid biosynthesis was blocked via suppression of the branch point enzyme chalcone synthase, and a substantial increase in raspberry ketone and its glycoside production was observed in tobacco flowers and leaves (Koeduka et al. 2021) (Figure 3). As with metabolic engineering in microbes, a strategy using multigene expression has been successful in flowering plants.

Notably, the RNAi-mediated suppression of the expression of a single gene often has a broader effect on the floral scent profile. For example, *p-coumarate 3-hydroxylase* (C3H), which catalyzes the formation of caffeoyl shikimate/quinate, a precursor of volatile phenylpropanoids, has been implicated in the protein–protein associations with phenylalanine ammonia-lyase, cinnamate-4-hydroxylase, and hydroxycinnamoyl-CoA:shikimate hydroxycinnamoyl transferase, which are key biosynthetic genes for the production of C_6_-C_1_ and C_6_-C_3_ volatile compounds. Downregulation of *C3H* in petunia resulted in lower levels of isoeugenol/eugenol, benzaldehyde, phenylacetaldehyde, and benzylbenzoate (Kim et al. 2019). Likewise, the levels of multiple floral volatiles were altered in transgenic petunia subjected to the RNAi-mediated suppression of the levels of thioesterase (Adebesin et al. 2018), cinnamate: CoA ligase (Klempien et al. 2012), caffeoyl shikimate esterase (Kim et al. 2023), cinnamoyl-CoA hydratase-dehydrogenase (Qualley et al. 2012), or 3-ketoacyl-CoA thiolase (Moerkercke et al. 2009). Therefore, the metabolic network of volatile floral compounds may be regulated by a system that is more complex than that previously thought.

## Conclusions

Over the past few decades, many biosynthetic genes and enzymes responsible for the synthesis of floral volatiles have been identified, and genetic engineering of these genes has led to significant progress in the modification of floral scents. Recent reports have evidenced that the glycosides of volatile compounds accumulate in flowers, an organ specialized for emission, in various plant species (Inagaki et al. 1995; Straubinger et al. 1999; Cna’ani et al. 2017). Furthermore, ATP-binding G transporters and lipid transfer proteins in petunia flowers induce the emission of volatile benzenoids/phenylpropanoids, wax loading, and cuticle formation, which affect the emission of volatile compounds in flower petals (Liao et al. 2023, 2021). Nevertheless, metabolic engineering often yields unpredictable results; therefore, plant metabolic networks, including the subcellular localization of biosynthetic enzymes, competitive pathways, and feedback regulation remain complex and elusive. Future research should focus on further biochemical characterizations of the molecules involved in intracellular accumulation of volatiles via glycosylation and their emission into the atmosphere. Additionally, a comprehensive understanding of the metabolic networks of floral scent profiles in terms of biosynthesis, accumulation, and transport is warranted for more efficient metabolic engineering of floral scents.

## Abbreviations

ABC transporter: ATP-binding cassette transporter
BAMT: benzoic acid carboxyl methyltransferase
BEBT: benzoyl-CoA:benzyl alcohol benzoyl transferase
BPBT: benzyl alcohol/phenylethanol benzoyl transferase
BSMT: benzoic acid/salicylic acid carboxyl methyltransferase
CFAT: coniferyl alcohol acyltransferase
DAHPS: 3-deoxy-D-arabino-heptulosonate-7-phosphate synthase
EOB: emission of benzenoids
IGS: isoeugenol synthase
MBMT: *p*-methoxybenzoic acid carboxyl methyltransferase
MYB: myeloblastosis
PAAS: phenylacetaldehyde synthase
PhC4H: *Petunia hybrida* cinnamate-4-hydroxylase
PhLHY: *Petunia hybrida* late elongated hypocotyl
PhpCAT: *Petunia hybrida* plastidial cationic amino-acid transporter
SAMT: salicylic acid carboxyl methyltransferase
